# Supplementary material for: The macaque ventral intraparietal functional connectivity patterns reveal an anterio-posterior specialization mirroring that described in human ventral intraparietal area
Source: Imaging Neurosci (Camb). 2025 Feb 27;3:imag_a_00491. doi: 10.1162/imag_a_00491 (PMC12319825; doi:10.1162/imag_a_00491)
Supplement: Supplementary Material [file imag_a_00491-supp.pdf]

The macaque ventral intraparietal functional connectivity patterns reveal an anterio-posterior specialization mirroring that described in human ventral intraparietal area

Supplemental data

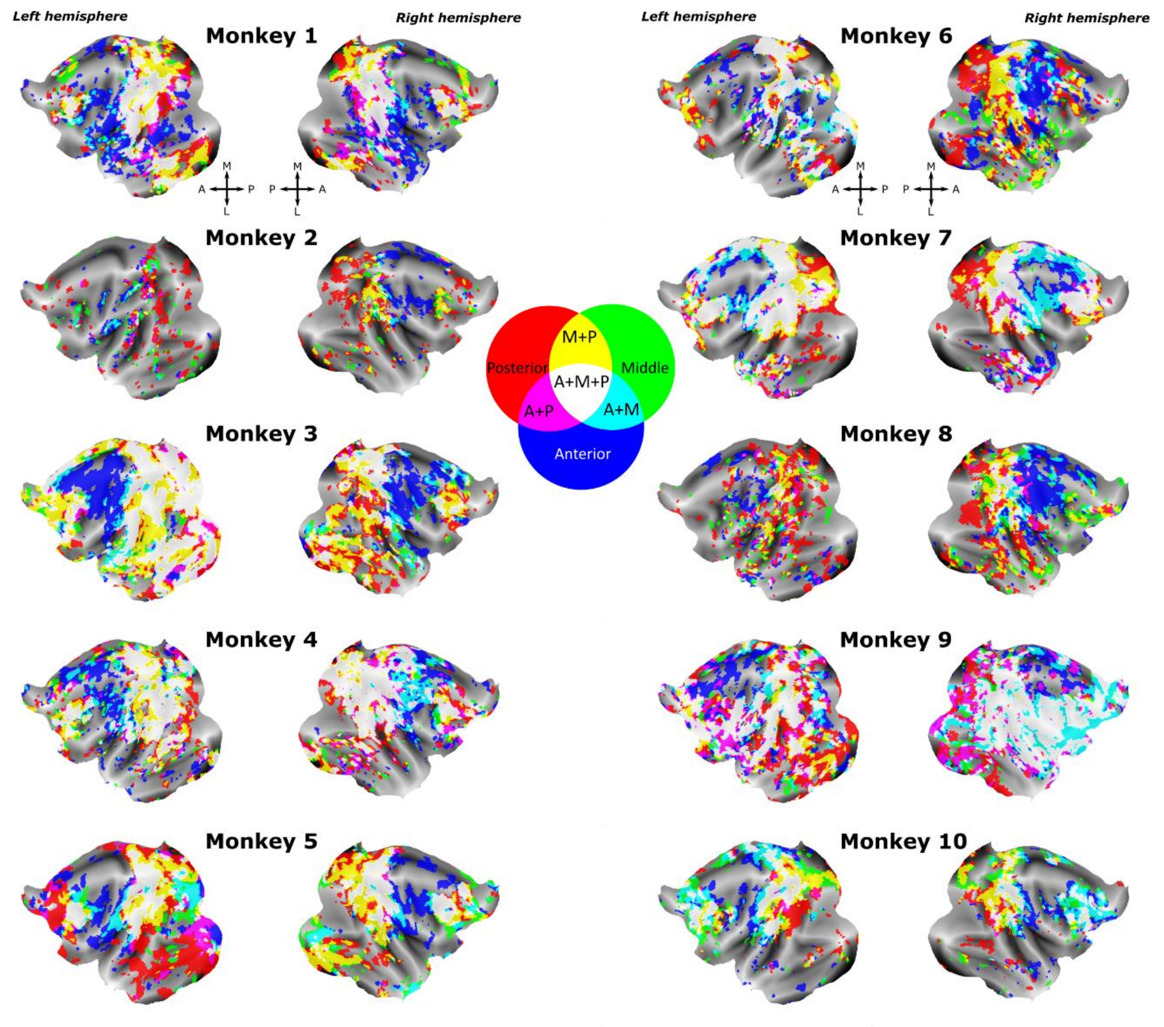

Supplementary Fig. S1 Topographical organization of the cortical regions that are uniquely functionally connected to one of the aVIP, mVIP and pVIP ROIs, or to two or more of these three ROIs in each of the ten individual monkeys. Flatmaps of both hemispheres show unique and overlapping regions for the three VIP connectivity maps. Surface voxels with z-scores above 0.05 are colored. Unique, non-overlapping voxels functionally connected to the anterior, middle and posterior VIPs are colored in blue, green and red, respectively. The anterior-middle, middle-posterior and anterior-posterior overlapping voxels are coded in cyan, yellow and magenta. Regions functionally connected to all three seeds are shown in white. AS, arcuate sulcus; CiS, cingulate sulcus; CS, central sulcus; IOS, inferior occipital sulcus; IPS, intraparietal sulcus; LS, lateral sulcus; LuS, lunate sulcus; OTS, occipitotemporal sulcus; PS, precentral sulcus; STS, superior temporal sulcus.

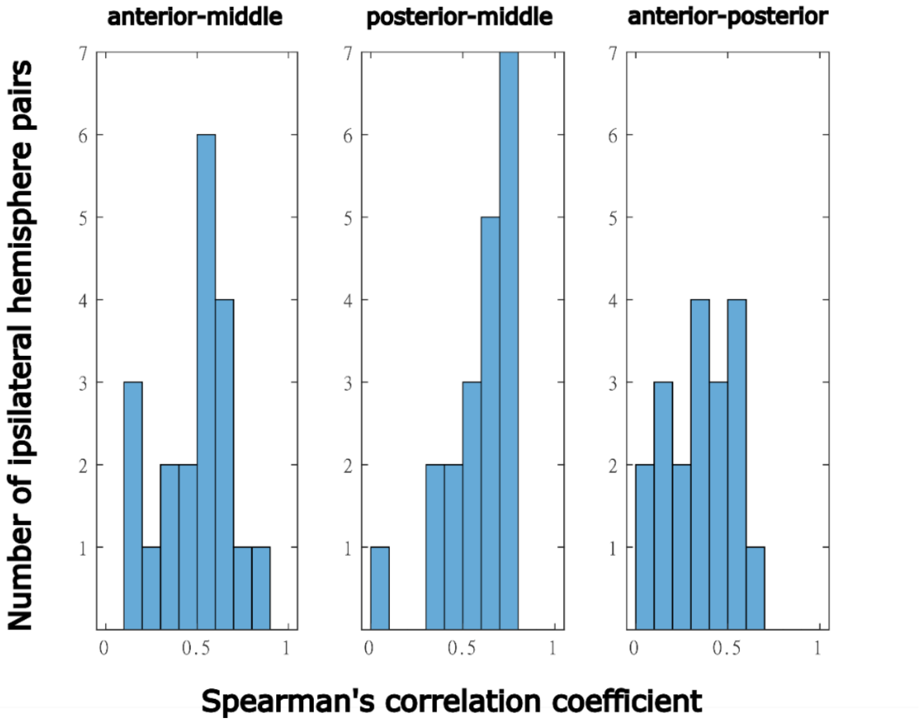

Supplementary Fig. S2 Distributions of spearman's correlation coefficient between the ipsilateral aVIP, mVIP and pVIP whole brain connectivity maps of the 10 individual monkeys. Spearman's correlation coefficient calculated between ipsilateral pairs of anterior-middle (aVIP-mVIP), posterior-middle (pVIP-mVIP) and anterior-posterior (aVIP-pVIP) VIP connectivity maps of each monkey, two hemispheres plotted together. All correlation coefficients have p values <0.05.

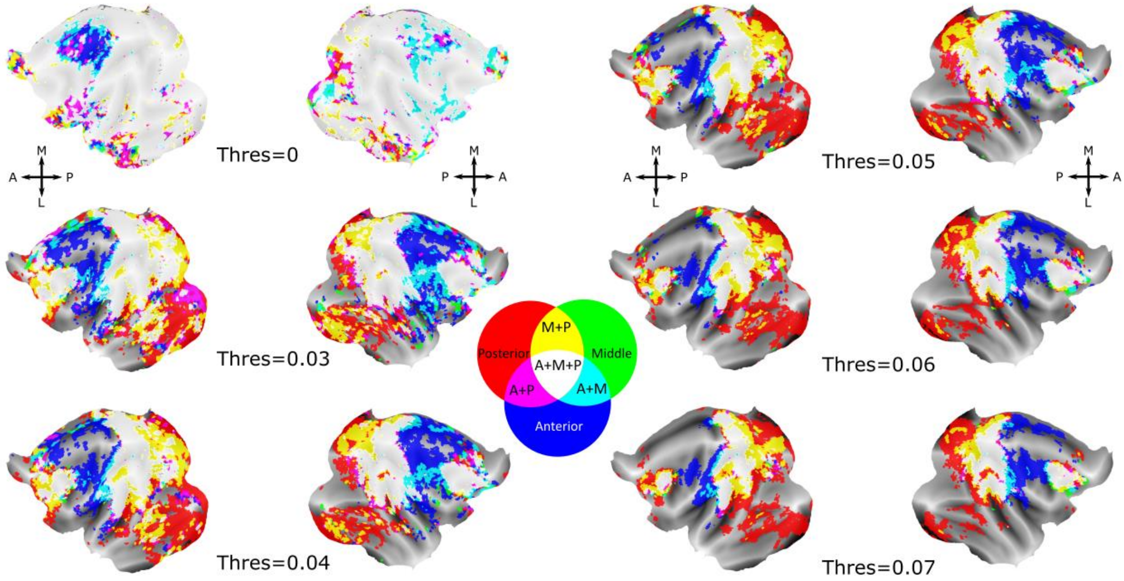

Supplementary Fig. S3 Different z-score thresholds to obtain topographical organization of the cortical regions that are uniquely functionally connected to one of aVIP, mVIP and pVIP ROIs, or to two or more of these three ROIs. Average flatmaps of both hemispheres show unique and overlapping regions for the three VIP connectivity map. Surface voxels with average z-scores above different thresholds (0 to 0.07) are colored. Unique, non-overlapping voxels functionally connected to the anterior, middle and posterior VIPs are colored in blue, green and red, respectively. The anterior-middle, middle-posterior and anterior-posterior overlapping voxels are in cyan, yellow and magenta. Regions functionally connected to all three seeds are in white. Pie charts show percentages of different categories of unique and overlapping surface voxels in the left and the right hemispheres with the same color code as in the flat maps. Black color in pie charts represents percentage of surface voxels without any signals.

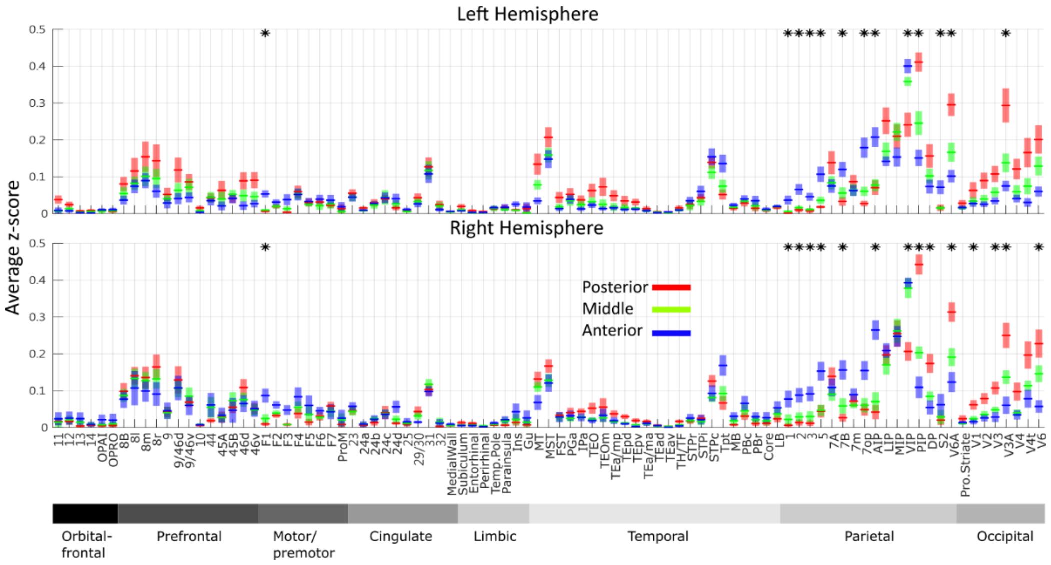

Supplementary Fig. S4. Average z-scores of functional correlation between aVIP, mVIP and pVIP in atlas-defined cortical functional regions and areas. For each area (Markov atlas, Markov et al., 2014), average z-scores of surface voxels correlated to aVIP (blue), mVIP (green) and pVIP (red) are calculated separately and plotted as line charts for the right and the left hemispheres separately. Shadows show standard error calculated across animals. Areas for which the average z-scores of surface voxels correlated to the three VIP seeds are significantly different (Kruskal-Wallis test with Benjamini-Hochberg procedure for multiple comparisons correction, $p<0.05$) are marked with a star on top. The areas are grouped by brain regions, in the following order: orbitofrontal, prefrontal, motor/premotor, cingulate, limbic, temporal, parietal and occipital. This arrangement allows most of the areas to be continuous in space with its previous and following areas in the figure.
